# Elevated carbon flux in deep waters of the South China Sea

**DOI:** 10.1038/s41598-018-37726-w

**Published:** 2019-02-06

**Authors:** Yung-Yen Shih, Hsi-Hsiang Lin, Dewang Li, Hsueh-Han Hsieh, Chin-Chang Hung, Chen-Tung Arthur Chen

**Affiliations:** 1 Department of Applied Science, R.O.C Naval Academy, Kaohsiung, 81345 Taiwan Republic of China; 2 Department of Oceanography and Asia-Pacific Ocean Research Center, National Sun Yat-sen University, Kaohsiung, 80424 Taiwan Republic of China; 3 Key Laboratory of Marine Ecosystem and Biogeochemistry, Second Institute of Oceanography, State Oceanic Administration, Hangzhou, 310012 PR China

## Abstract

We measured particulate organic carbon (POC) fluxes from the euphotic zone into the twilight zone and deep waters (>1000 m) that occurred between the shelf and the basin in the South China Sea (SCS) and at the SouthEast Asia Time Series Station (SEATS) using floating sediment trap arrays. Additionally, selected sinking particles were imaged by scanning electron microscope (SEM) to reveal particle morphology and composition. Results showed large variations in POC fluxes with elevated values (32–104 mg-C m^−2^ d^−1^) below the euphotic zone and a trend towards lower values in the deep SCS. Vertical POC fluxes measured in deep waters between the shelf and the SCS basin were much higher than those estimated by Martin’s attenuation equation. These elevated POC fluxes in deep waters were attributed to lateral particle transport as opposed to enhanced settling out of the euphotic zone. SEM images of sinking particles at 150 m show abundant marine biogenic detritus, while those in deep waters contained a higher proportion of lithogenic material. A great deal of the spatial variability in POC fluxes across the twilight zone and deep waters of the SCS cannot be represented by current biogeochemical models.

## Introduction

The export of particulate organic carbon (POC) transfers anthropogenic carbon dioxide into the ocean interior^1^, and provides energy to the ocean food web^2,3^. Such carbon transport is often simplified to one-dimensional vertical process due to the dominant of sinking POC flux^4,5^. It is believed that the carbon budget in the deep ocean is maintained at steady state by a balance between POC supply and metabolic activity. The Martin’s flux attenuation equation: POC flux = Flux_100_ (Z/100)^−b^, where b is the rate constant of POC flux attenuation, z is the trap depth, and Flux_100_ is the POC flux at a reference depth of 100 m or other depths (e.g. 120 or 150 m), is widely used to estimate downward POC flux at different depths^6^. However, some researchers have questioned the applicability of this flux equation because of discrepancies between observed and modeled carbon fluxes in deep waters. They have suggested that the POC flux attenuation may be controlled by the magnitude of the flux, the characteristics of sinking particles and the specifics of sampling locations^7,8^. Recently, Burd *et al*.^9^ reviewed numerous field studies and found that estimated metabolic activity in deep waters seemed to be higher than organic matter flux. The discrepancy between carbon fluxes and metabolic activity in the deep ocean can be as much as two orders of magnitude. Burd *et al*.^9^ concluded that the imbalance between sources and sinks was poorly understood, but that it pointed to lateral particle transport and slowly sinking particles as two important yet poorly quantified terms.

The South China Sea (SCS) is the largest marginal sea in the world and it has a complicated flow: with the surface waters of the Kuroshio flowing into the SCS and the surface water of the SCS flowing out to the Taiwan Strait and the Luzon Strait^10^. Chen^11^ noted an eastward SCS intermediate water (SCSIW) outflow (an average flow rate equals to 1.9 Sv (10^6^ m^3^ s^−1^), at depths from 350 to 1350 m) into the western Philippine Sea suggesting that SCSIW was an important water mass carrying nutrients to the East China Sea (ECS) shelves and seas of south Japan.

The oligotrophic ocean of the northern South China Sea (NSCS), far from the influence of land-runoff, shows open ocean characteristics with a POC flux at the euphotic zone ranging from 32 to 73 mg-C m^−2^ d^−1^ in summer^12^, which is much less than the eutrophic water in the southern ECS (58–785 mg-C m^−2^ d^−1^)^13^. POC inputs into the oligotrophic waters of the SCS vary seasonally^14^. Besides, several transport mechanisms can carry organic carbon into deep waters in the SCS including internal waves-triggered organic carbon export^15^, eddies-driven organic matter flux^16^, etc. Moreover, a significant lateral transport of sedimentary carbon into the deep NSCS is thought to be occurring^17^ although this has yet to be confirmed. Frequent typhoons^18,19^, eddies^20^, earthquakes^21^ and a steep topography in the NSCS all facilitate the lateral transport of biogenic and terrestrial carbon across the shelf^22,23^. This transport may also occur sporadically between the continental shelf and deep ocean basin but data about this phenomenon is very scarce.

In this study, we measured carbon fluxes from upper layers (150 m), twilight zones (150 to 1000 m) to deep waters (greater than 1000 m) at the border between the shelf and the basin, and in deep waters in the SCS using our floating sediment trap array. We estimated POC fluxes, measured trace metal concentrations and obtained scanning electron microscope (SEM) images of the sinking particles. We then attempted to discuss the origin and processes of the laterally transported POC observed in the boundary between the shelf area and the open ocean of the SCS.

## Results

### Distributions of chlorophyll *a* and POC

**Figure 1:**
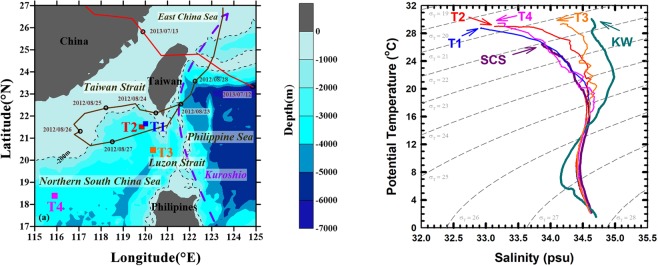
Sampling locations of the sediment trap in the northern South China Sea (**a**) andT-S diagrams at stations T1, T2, T3, T4, SCS (South China Sea) and KW (Kuroshio Water) (**b**). Stations T1, T2, T3 and T4 are denoted by blue, red, orange and pink square symbols. Station T4 (for comparison) was located in the central basin. The tracking information of typhoon *Tembin* (Aug. 22–29, 2012) and *Soulik* (Jul. 11–13, 2013) are shown by brown and red solid lines.

**Figure 2:**
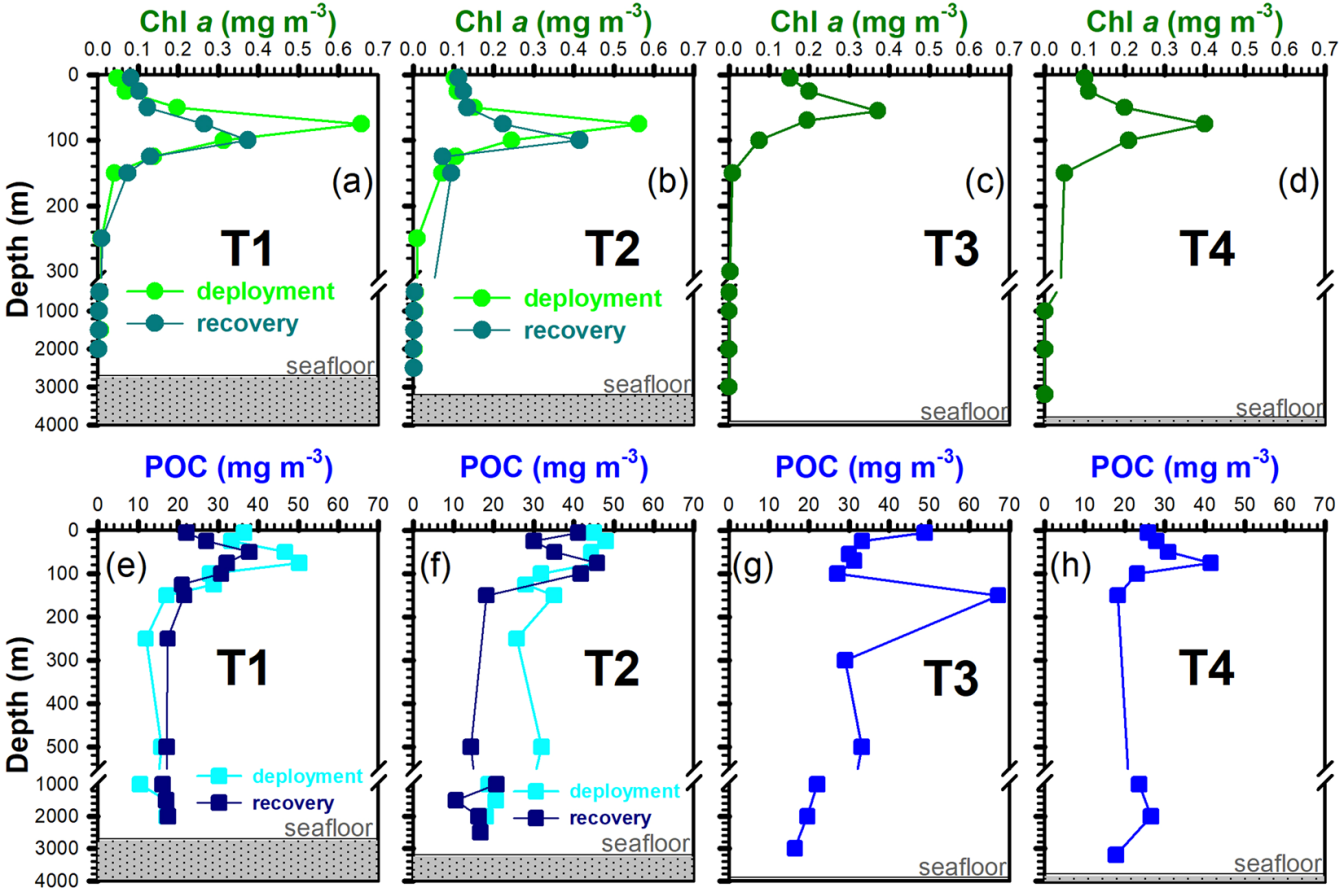
Vertical distributions of Chl *a* and POC concentrations at stations T1 (**a**,**e**), T2 (**b**,**f**), T3 (**c**,**g**) and T4 (**d**,**h**), respectively. Chl *a* and POC concentrations after the deployment and before the recovery of the trap at stations T1 and T2 are shown in (**a**,**b**,**e**,**f**).

A diagram of potential temperature (T) against salinity (S) is shown in Fig. 1. It is worth noting that the water characteristics of stations (Sts.) T1, T2, T3 and T4 appear to result from the mixing between the Kuroshio and the SCS^24^. Distributions of Chl *a* concentrations at all stations showed a subsurface Chl *a* maximum at 75–100 m, below which Chl *a* decreased with depth to a background value (<0.1 mg m^−3^) deeper than 200 m (Fig. 2). The vertical distributions of POC concentrations at the four stations were similar to the Chl *a* pattern, with a subsurface maximum suggesting biogenic sources of POC. However, elevated POC concentrations (30~70 mg m^−3^) were observed in the twilight zone (~150 to 500 m) at Sts. T2 and T3, perhaps due to the more recalcitrant nature of the particles in the twilight zone. Elevated POC concentrations were also observed in deep waters at Sts. T1 and T2 (~30 mg m^−3^) when compared to background values (~20 mg m^−3^) at adjacent deep-water sites (Fig. 2). Resuspension of material settled at Sts. T1 and T2 is unlikely since the deepest trap were at 1000 and 2000 m, respectively, while water depths were ~2700 and ~3200 m, respectively (Fig. 2). In addition, the sampling location was approximately 100 km away from Taiwan where the influences of terrestrial materials are expected to be insignificant during non-typhoon conditions.

### POC fluxes in the northern SCS

**Figure 3:**
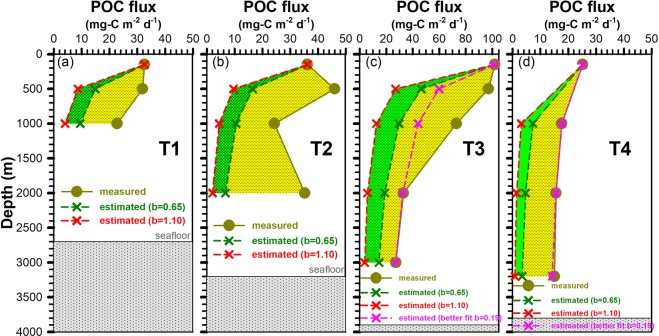
POC fluxes at stations T1 (**a**), T2 (**b**), T3 (**c**) and T4 (**d**), respectively. Measured and estimated POC fluxes are shown as solid circles and lines, and cross symbols and dashed lines, respectively. Estimated POC fluxes were calculated using the b values 0.65^25^, 1.10^28^ and better fits (0.44 and 0.19 at T3 and T4, respectively) according to the Martin’s equation^6^. Dashed green and yellow areas represent a positive anomaly in terms of the mass of POC transported per meter per day.

POC export fluxes from the euphotic zone (i.e. 150 m) were approximately 33 (St. T1) to 36 mg-C m^−2^ d^−1^ (St. T2), which is slightly lower than the value (50 ± 7 mg-C m^−2^ d^−1^) reported for the NSCS^25^ as a whole. Interestingly, elevated POC fluxes (32 and 23 mg-C m^−2^ d^−1^ at 500 and 1000 m) were found at St. T1 which did not exponentially decrease with increasing depth based on Martin’s attenuation curve (Fig. 3a). A similar trend of elevated POC fluxes (46, 24 and 35 mg-C m^−2^ d^−1^ at 500, 1000 and 2000 m) was also observed at St. T2 (Fig. 3b). An analogous distribution profile of POC fluxes was observed at St. T3 (Fig. 3c) with 102, 97, 73, 33 and 27 mg-C m^−2^ d^−1^ at 150, 500, 1000, 2000 and 3000 m, respectively.

However, POC fluxes at St. T4 (i.e. SouthEast Asia Time Series Station, SEATS) decreased smoothly with increasing depth (25, 18, 16, 15 mg-C m^−2^ d^−1^ at 150, 1000, 2000 and 3200 m) (Fig. 3d). Although lower than values at Sts. T1~T3, these values were slightly higher than fluxes previously measured at SEATS when particles were collected by moored sediment traps (deployed for six months or twelve months)^23^. Our elevated fluxes (collected time ~1 day) at St. T4 are reasonable since sinking particles collected by moored traps (collected time: ~six months) will be degraded, which can be inferred from Hung *et al*.^26^ assessment about the extent of degradation of sinking particles collected by sediment traps.

The distribution of POC concentrations in deep waters at Sts.T2 and T3 (Fig. 2) showed anomalously high values situated 2500 m above the seafloor suggesting the trap-collected particles from deep waters were less likely affected by resuspended bottom sediments and potential lateral POC transport occurring at Sts. T2 and T3. Similar lateral transport of sinking material is reported at the depth of 500 to 1500 m in the southern ECS^27^.

**Figure 4:**
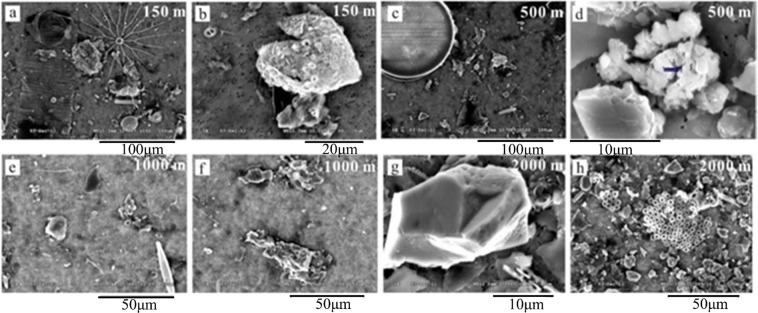
SEM images of sinking particles collected from sediment traps at 150 (**a**,**b**), 500 (**c**,**d**), 1000 (**e**,**f**) and 2000 m (**g**,**h**) of station T2., (**a**) biogenic detritus, degraded diatom (i.e. *Bacteriastrum spp*.) cells (**b**) fecal pellets containing degraded coccolithophore cells (**c**) biogenic detritus, degraded diatom (i.e. *Coscinodiscus spp*.) cells (**d**) lithogenic detritus containing manganeseoxides (**e**) small biogenic detritus, degraded diatom (i.e. *Navicula spp*.) cells (**f**) small biogenic detritus and fecal pellets (**g**) lithogenic detritus (**h**) small biogenic detritus and aggregates.

**Figure 5:**
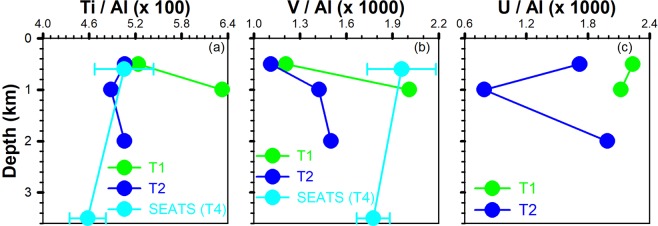
Vertical ratios of Ti/ Al and V/Al (**a**,**b**) in sinking particles at T1, T2 and SEATS (Tables 1, 2 and 3 of Ho *et al*.^50^) (i.e. T4) sites. (**c**) vertical distributions of U/Al at T1 and T2.

Figure 4 shows SEM images of bulk sinking particles collected from depths of 150, 500, 1000 and 2000 m at St. T2. The composition and size-classes of these particles cannot be quantified from the images, but one can clearly see the presence of diatom cells, fecal pellets, detritus and decomposed coccolithophore cells at 150 m. An increasing proportion of non-biogenic material was observed in deep waters (i.e. water depth >500 m) (Fig. 4). Moreover, the Al-based lithogenic flux increased with depths in the middle layer (Fig. 5). The results show that both biogenic and non-biogenic particles contributed to the POC flux in deep waters.

## Discussion

### Carbon fluxes in deep waters

Under normal conditions, the POC flux decreases with increasing depth either through its decomposition to dissolved inorganic carbon (DIC) or its conversion to dissolved organic carbon (DOC)^1^. In this study, however, we found elevated POC fluxes below 500 m which provide evidence for the large-scale lateral advection thought to occur in these deep waters. The measured carbon export fluxes to the deep waters at Sts. T1 and T2 were approximately 23–32 and 24–46 mg-C m^−2^ d^−1^, respectively.

It is common practice to predict the attenuation of POC flux with increasing depth using Martin’s equation^6^. We used b values previously measured in the NSCS^25,28^ of 0.65 (lower value) and 1.10 (upper value) to estimate the POC flux attenuation. This calculation led to POC fluxes of 15–9 and 9–4 mg-C m^−2^ d^−1^ at water depth of 500 and 1000 m at St. T1, and 17–10, 11–5 and 7–2 mg-C m^−2^ d^−1^ at water depth of 500, 1000 and 2000 m at St. T2, respectively (Fig. 3). In addition to using the b values described above, we also estimated POC fluxes at Sts. T3 and T4 using fitted coefficients which turned out to be b = 0.44 at St. T3 and b = 0.19 at St. T4. The overall results revealed that estimated POC fluxes were 60–27, 44–13, 32–6 and 27-4 mg-C m^−2^ d^−1^ at water depths of 500, 1000, 2000 and 3000 m at St. T3, and 18–3, 16–1 and 14–1 mg-C m^−2^ d^−1^ at water depth of 1000, 2000 and 3200 m at St. T4, respectively. At Sts. T1, T2 and T3, the POC deep water fluxes measured by the sediment traps were significantly higher than any of the values calculated from Martin’s equation. This supports the view that lateral transport of sediment particles contributed to elevated POC fluxes in deep waters. Alternatively, it may be that the POC flux attenuation at Sts. T3 and T4 did not abide by Martin’s equation as a result of especially efficient carbon transfer to depth (Fig. 3). Indeed, similar findings have been reported at K2 site (located in the Northwest Pacific subarctic gyre) with an elevated transfer efficiency of sinking POC in the twilight zone^8^. Most importantly, this large difference between measured and estimated values implies that POC fluxes in deep waters of large open marginal seas, such as the SCS, cannot be adequately described by a traditional particle attenuation function (e.g. Martin’s equation) under events of lateral transport.

According to Hung and Gong^29^ and Hung *et al*.^30^, the e-ratio (POC flux/primary production (PP)) in open ocean water is around 0.1 (0.05~0.16). If we use e-ratio 16% (maximum value) and previous annual PP values (280 mg-C m^−2^ d^−1^)^14^, the estimated POC fluxes (b = 0.65 and 1.10)^25,28^ at 500 and 1000 m would be 12–20 and 6–13 mg-C m^−2^ d^−1^, respectively. The estimated POC fluxes are much lower than observed values (32–46 and 23–24 mg-C m^−2^ d^−1^ at 500 and 1000 m, respectively) suggesting POC fluxes in deep waters cannot be accounted for by biogenic particles settling from the upper ocean.

The average POC fluxes in deep waters were 27 and 35 mg C m^−2^ d^−1^ at Sts. T1 and T2, respectively. Assuming the affected area was 10^6^ m^2^ (=100 km × 10 m, based on the steep topography in the NSCS) during each episodic event, it follows that the integrated vertical flux of POC in deep waters at Sts. T1 and T2 was 27 × 10^3^ g-C d^−1^ (on September 5^th^, 2012) and 35 × 10^3^ g-C d^−1^ (September 26^th^, 2012). The lateral POC flux can be estimated to be 28 × 10^8^ g-C d^−1^ (=POC concentration (~17 mg-C m^−3^) at 1000 m × average flow rate at 1000 m) if we assume an eastward flow of 1.9 Sv (m^3^ s^−1^) at depth of 1000 m^11^. Hence, the lateral POC flux is several orders of magnitude higher than the integrated vertical flux estimated by sediment traps. It is worth noting, however, that this lateral transport likely occurs sporadically, when currents strong enough to resuspend settled material contact the continental slope seafloor. Such resuspension events may not last for the full 24 h period implied by our calculation. More studies are clearly needed to better understand real lateral carbon transport in deep waters in the SCS.

We have good reasons to believe that some POC can be transported to the ECS and on to the western Pacific via the eastward flowing SCSIW, and that this transport occurs at depths of 350 to 1300 m^11^. Other deep lateral POC flux can be stored in deep waters (>1300 m), potentially trapping the carbon for centuries, if not millennia, given the long residence time of the deep seawater^31^. In this way, the lateral carbon transport plays an important role for carbon sequestering in deep waters of the NSCS.

### Possible mechanisms affecting lateral advection

**Figure 6:**
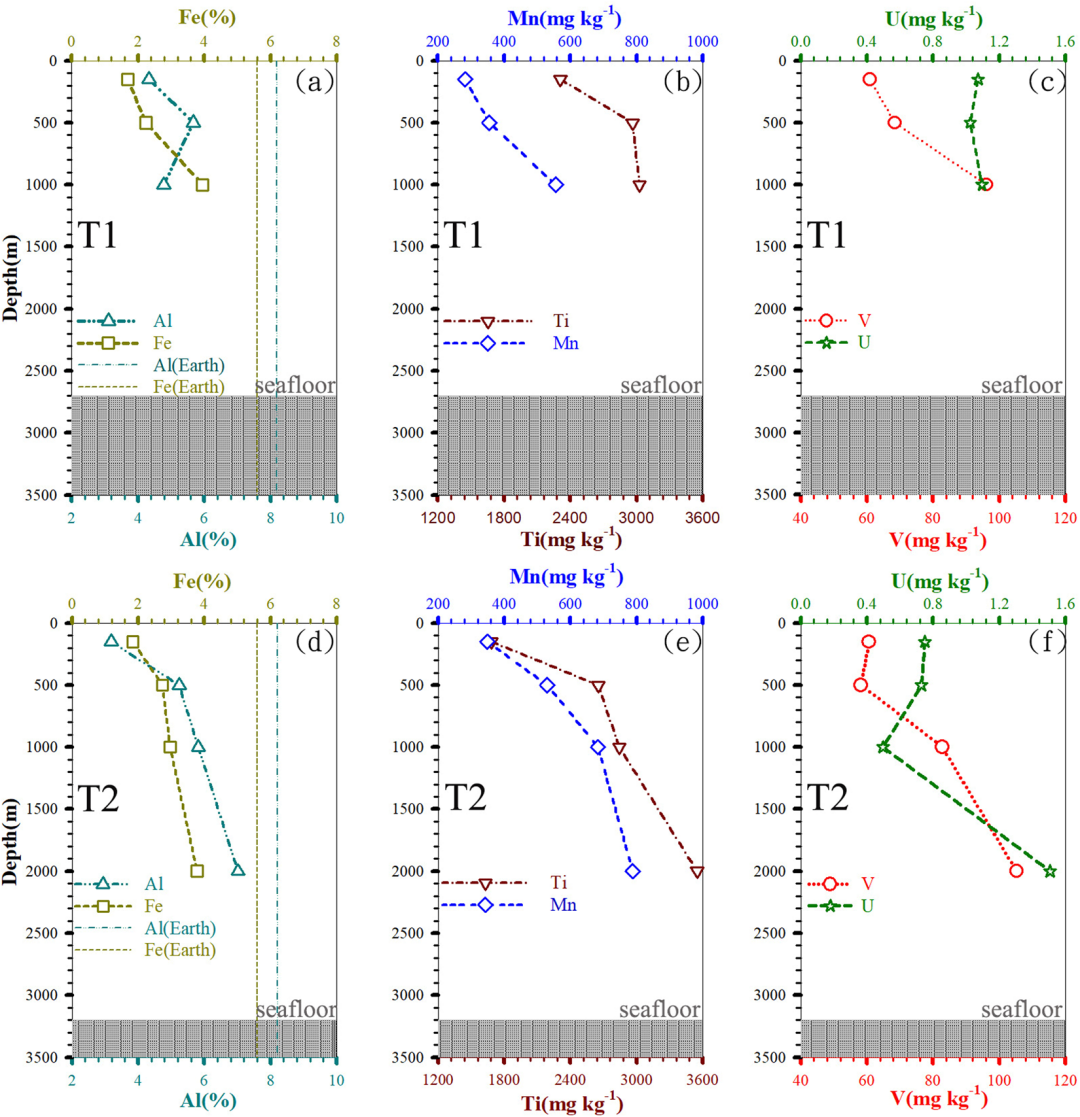
Vertical distributions of {Al, Fe (**a**,**d**), Mn, Ti (**b**,**e**), V and U (**c**,**f**)} in sinking particles collected by sediment trap at stations T1 and T2, respectively.

Several mechanisms, including typhoon-induced hyperpycnal currents^32^, mesoscale eddies^33^, earthquakes^34^ and episodic events^28,35^ such as “benthic storms” associated with current interaction with steep topography, may contribute to lateral transport in the SCS. For example, the funnel-like topography of a canyon off southwest Taiwan induces sediment transport that is mainly driven by flood events in the summer and tidal action in the winter season^32^. Part of our sampling was undertaken in September, i.e. during the typhoon season. Under those conditions, large quantities of suspended riverine material containing some lithogenic particles mixed with biogenic particles are delivered directly into the deep sea by high-energy rivers^22^. Our measurements showed the Al-normalized flux (Fig. 5) as well as metal concentrations (Fe, Al, Mn, Ti, U and V) (Fig. 6) increasing with increasing depth. Moreover, percentages of Fe and Al in sinking particles below 500 m were close to those in the earth crust^36^, indicating that sinking particles in the deep waters were partially from terrestrial sources^37^ (Fig. 6). Indeed, many researchers also reported that typhoon-induced floods and hyperpycnal flows play a key role in the transport of terrestrial materials and carbon to the ocean^23,38,39^. Mulder *et al*.^40^ described that hyperpycnal processes involve transporting river material, do not entrain seafloor sediment and directly transport to the marine environment by a turbulent flow (hyperpycnal turbidity current). Typhoons are not only physical agents for enhanced delivery of terrestrial material to the sea. Geological agents can also trigger gravity flows such as turbidity currents that eventually leave deposits on the seafloor and form large-scale deep-water depositional systems^40,41^. For instance, typhoon *Tembin* was a category-2 typhoon (sustained winds: 30 m s^−1^) which traveled at a speed of 3.0~5.5 m s^−1^ from 22–29 August, 2012. The typhoon affected the study area (Sts. T1 and T2) for several days and dumped high amounts of rainfall, peaking at ~580 mm in Taiwan and resulted in flooding in southern Taiwan (a similar event was also recorded for typhoon *Soulik*, 11–13 July, 2013). It is likely that typhoon-induced hyperpycnal turbidity currents drive lateral particle transport from the shelf to basin area in the SCS. The lower salinity water (blue curve in Fig. 1b) found in the upper waters of St. T1 about 9–10 days after the passage of typhoon *Tembin* attests to the amount of rainfall and flooding that took place. Similar typhoon-induced heavy rainfall and flooding brought to open ocean after typhoons have been reported by Lee *et al*.^42^ and Hung *et al*.^43^.

Furthermore, frequent earthquakes may be another trigger factor facilitating lateral POC transport^21^. For example, it is summarized that earthquakes often reinforce the effect of typhoons and vice versa, thus substantially enhancing the sediment load in fluvial systems^44,45^, which may frequently increase hyperpycnal concentrations. But it is difficult to estimate its real impact since the seas around Taiwan have more 10,000 earthquakes per year (www.cwb.gov.tw).

Some researchers also reported that mesoscale eddies can carry particles from nearshore to offshore^20,33^. The mesoscale eddies can even carry materials to the open ocean, as evidenced by satellite image observations, but it is not evident that these materials can enter deep waters. We cannot definitely distinguish all factors based on current data set, since resuspended particles may occur in the coast or on the continental shelf, and then be re-located elsewhere due to episodic events. Therefore, the elevated POC fluxes in deep waters probably can be caused by typhoons, earthquakes, eddies, seasonal and interannual variability in currents and/or cooling effects as well as deep layers sediment resuspension^28,33,35,46^. One may ask whether the deep sea POC flux is unique to the northern South China Sea or whether it applies to the whole South China Sea. For example, Chen *et al*.^10,11^ used a box model to show that the eastward flow of the SCSIW (350~1300 m) can transport high-nutrient waters across the entire ECS and beyond. Lateral POC fluxes and POC concentrations could be measured directly but this would require intensive sampling and lots of ship time. Until now, very few cruises have captured lateral POC transport in the SCS, thus we are not sure if the lateral contribution to the POC export flux is present in all parts of the deep (>500 m) SCS. Such deep water lateral transport in the SCS definitely requires further study.

## Materials and Methods

Four cruises aboard the R/V *Ocean Researcher III* (*OR-III*) and *V* (*OR-V*) were conducted in each of the following periods: Sep. 5–7 (T1), Sep. 25–28 (T2), 2012 and Jul. 14–17 (T3) and Sep. 12–18 (T4, *OR-V*), 2013. The first cruise was conducted approximately a week after a category 2 typhoon *Tembin*, and the third cruise was conducted ~2 days after typhoon *Soulik* (category 2). Sinking particles were collected at 150, 500 and 1000 m at St.T1 (21.7°N 120.0°E, water depth ~2700 m) located at the boundary between the shelf and the basin using a drifting sediment trap array^13,25^. At the second cruise, the sediment trap was also deployed at the boundary between the shelf and the basin (St. T2, 21.6°N 119.9°E, water depth ~3200 m) with four traps at 150, 500, 1000 and 2000 m, respectively. During two additional cruises, sinking particles were collected from deep waters (St. T3, 20.3°N 120.3°E, water depth ~3900 m; St. T4, 18.0°N 116.0°E, water depth ~3800 m) by using the same approach with depths of 150, 500, 1000, 2000 and 3000 m on Jul. 14–17; with depths of 150, 1000, 2000 and 3200 on Sept. 12–18, 2013, respectively (Figs 1 and 3). The deployment period was approximately 24 to 36 hours (less than 24 hours if bad weather conditions occurred). Swimmers on the filter were carefully removed using forceps under a microscope. POC flux measurement was determined according to Hung *et al*.^25^.

A SBE 9/11 plus CTD sensor was used to assess hydrographic conditions. Seawater samples were collected to measure POC and Chl *a* concentrations. Organic carbon in suspended as well as sinking (trap collected) materials were assessed based on Hung *et al*.^25^ using an elemental analyzer (Elementa, Vario EL-III, Germany). Chl *a* was analyzed with a Turner Designs 10-AU-005 fluorometer after extraction with 90% acetone following by the non-acidification method^47^. Contents of metal elements in sinking particles were determined by quadrupole-based inductively coupled plasma mass spectrometer (Hsu *et al*.^48^) after total digestion of the particles with a combination of HF, HNO_3_ and HClO_4_. Selected bulk sinking particles were filtered on polycarbonate filters and imaged by scanning electron microscopy (SEM) according to Hung *et al*.^49^.
